# mTOR inhibitor improves autistic-like behaviors related to *Tsc2* haploinsufficiency but not following developmental status epilepticus

**DOI:** 10.1186/s11689-021-09357-2

**Published:** 2021-04-17

**Authors:** Tomas Petrasek, Iveta Vojtechova, Ondrej Klovrza, Klara Tuckova, Cestmir Vejmola, Jakub Rak, Anna Sulakova, Daniel Kaping, Nadine Bernhardt, Petrus J. de Vries, Jakub Otahal, Robert Waltereit

**Affiliations:** 1 National Institute of Mental Health, Topolova 748, 250 67 Klecany, Czech Republic; 2 First Faculty of Medicine, Charles University, Prague, Czech Republic; 3 Second Faculty of Medicine, Charles University, Prague, Czech Republic; 4 Faculty of Science, Charles University, Prague, Czech Republic; 5 Department of Psychiatry, University Hospital and Medical Faculty Carl Gustav Carus, Technical University of Dresden, Dresden, Germany; 6 Division of Child & Adolescent Psychiatry, University of Cape Town, Cape Town, South Africa; 7 Department of Developmental Epileptology, Institute of Physiology CAS, Prague, Czech Republic; 8 Department of Child and Adolescent Psychiatry, University Hospital and Medical Faculty Carl Gustav Carus, Technical University of Dresden, Dresden, Germany; 9 Department of Child and Adolescent Psychiatry, University Medical Center Göttingen, Von-Siebold-Str. 5, 37075 Göttingen, Germany

**Keywords:** Tuberous sclerosis complex, TSC, Autism spectrum disorders, Developmental status epilepticus, mTOR, Everolimus

## Abstract

**Background:**

Tuberous sclerosis complex (TSC), a multi-system genetic disorder often associated with autism spectrum disorder (ASD), is caused by mutations of *TSC1* or *TSC2*, which lead to constitutive overactivation of mammalian target of rapamycin (mTOR). In several *Tsc1+/-* and *Tsc2+/-* animal models, cognitive and social behavior deficits were reversed by mTOR inhibitors. However, phase II studies have not shown amelioration of ASD and cognitive deficits in individuals with TSC during mTOR inhibitor therapy. We asked here if developmental epilepsy, common in the majority of individuals with TSC but absent in most animal models, could explain the discrepancy.

**Methods:**

At postnatal day P12, developmental status epilepticus (DSE) was induced in male *Tsc2+/-* (Eker) and wild-type rats, establishing four experimental groups including controls. In adult animals (*n* = 36), the behavior was assessed in the paradigms of social interaction test, elevated plus-maze, light-dark test, Y-maze, and novel object recognition. The testing was carried out before medication (T1), during a 2-week treatment with the mTOR inhibitor everolimus (T2) and after an 8-week washing-out (T3). Electroencephalographic (EEG) activity was recorded in a separate set of animals (*n* = 18).

**Results:**

Both *Tsc2+/-* mutation and DSE caused social behavior deficits and epileptiform EEG abnormalities (T1). Everolimus led to a persistent improvement of the social deficit induced by *Tsc2+/-*, while deficits related to DSE did not respond to everolimus (T2, T3).

**Conclusions:**

These findings may contribute to an explanation why ASD symptoms in individuals with TSC, where comorbid early-onset epilepsy is common, were not reliably ameliorated by mTOR inhibitors in clinical studies.

**Supplementary Information:**

The online version contains supplementary material available at 10.1186/s11689-021-09357-2.

## Background

Autism spectrum disorder (ASD) is a neurodevelopmental disorder occurring in about 1% of the general population and well recognized as a global public health concern [1]. Epilepsy is present in about 21% of individuals with ASD who also have an intellectual disability and in about 8% of those without intellectual disability. Epilepsy is associated with a range of problem behaviors and significant treatment challenges [2–5]. However, there is still a very limited understanding of the fundamental links between ASD and seizures and a dearth of pre-clinical and clinical intervention studies of ASD in the context of epilepsy.

Tuberous sclerosis complex (TSC) is an autosomal dominant disorder with an incidence of 1:6000, with manifestations (including, but not limited to, benign tumors and other types of lesions) that can affect almost every organ in the body, including the brain. TSC is associated with ASD in up to 50% of individuals, and in turn, accounts for 1–4% of overall autism cases [6–8]. A lifetime history of epilepsy is reported in 70–90% of individuals with the disorder [9–14].

TSC is caused by mutations in either *TSC1* or *TSC2* resulting in dysfunction of the TSC1-TSC2 intracellular protein complex, causing overactivation of the mTOR signaling pathway [15]. The pharmacological class of mTOR inhibitors (mTORi) has emerged as molecularly targeted treatments for TSC1-TSC2-protein complex overactivation in TSC. For several organ manifestations in TSC, including renal angiomyolipoma, subependymal giant cell astrocytoma (SEGA), and as adjunctive treatment in treatment-resistent epilepsy, mTORi are now FDA- and EMA-approved therapies [16–19].

Apart from ASD, TSC is associated with a wide range of TSC-associated neuropsychiatric disorders (TAND) seen in up to 90% of individuals with TSC [15]. There is therefore significant interest in understanding the molecular (and other) mechanisms of TAND in order to identify appropriate treatments. After earlier proposals that ASD in TSC was caused by structural and/or electrophysiological aberrations, de Vries and Howe [20] proposed that direct molecular pathways may be sufficient to lead to ASD and other TAND deficits, and that mTORi may therefore be molecular-targeted treatments [20].

In *Tsc1* and *Tsc2* animal models, mTORi were shown to reverse cognitive [21] and social behavior impairments [22, 23], and early-phase mTORi in adults with TSC suggested potential improvement in memory and executive skills [24]. However, in subsequent trials of children and adolescents, two phase II trials reported no significant improvement in TAND manifestations including intellectual ability, behavioral problems, or ASD-related symptoms, after 6–12 months of mTORi administration [25, 26].

Epilepsy is seen in at least 70% of individuals with TSC. An association between early seizure onset (especially infantile spasms during the first year of life) and poor neurodevelopmental outcome, including ASD, has been consistently reported [5, 7, 27–36]. It is therefore of potential importance that reversal of cognitive and social behavior deficits by mTORi was mostly observed in studies conducted in *Tsc1+/-* and *Tsc2+/-* mice without an additional epilepsy model [21–23]. By contrast, the majority of TSC patients enrolled in the phase II mTORi studies also had a history of epilepsy [25, 26].

We previously reported social deficits in adult Eker rats (a spontaneous *Tsc2* haploinsufficiency model) reminiscent of the findings in *Tsc1+/-* and *Tsc2+/-* mice. We also observed social deficits induced by early developmental status epilepticus (DSE) in wild-type rats, and reported additional social deficits in *Tsc2+/-* rats when combined with DSE [37–39]. The combination of *Tsc2+/-* and DSE corresponds more closely to the situation of the majority of individuals with TSC plus ASD. Recently, we reported that social deficits in the combined *Tsc2+/-* plus DSE model responded to the mTORi everolimus [40]. However, whether the autistic-like behaviors induced by *Tsc2+/-*, by DSE, or by combination of TSC and DSE have differential responsiveness to mTORi has not been examined to date.

Taking together the clinical observations and our experimental animal work, we hypothesized that ASD phenotypes in TSC resulted from a combination of TSC1/TSC2 molecular deficits (directly leading to some social deficits) and additional social impairments caused by seizures. We proposed that the direct TSC molecular deficits may be sensitive to mTORi, but that seizure-related deficits may not be, thus explaining why TAND manifestations in individuals with TSC may have been unresponsive to mTORi. Here, we studied the effect of everolimus, an mTORi, on the social deficits induced by the two factors (*Tsc2+/-* and DSE), either in isolation or in combination.

## Methods

### Animals

Rats used in this study were heterozygous *Tsc2+/-* (Eker) males, RRID:RGD_625624 (homozygous mutants are not viable) and their wild-type Long-Evans littermates, bred at the National Institute of Mental Health (NIMH) in Klecany, Czech Republic. Breeding *Tsc2+/-* males originated from the breeding colony at Technische Universität Dresden, Germany, or from subsequent generations born at NIMH. Long-Evans dams, RRID:RGD_2308852, were purchased from a supplier (Charles River, Germany). We took care to use wild-type females only, as dam genotype was demonstrated to influence maternal care and pup behavior in *Tsc2+/-* mice [41]. Breeding was performed in individually ventilated cages (Tecniplast, 40×35×21 cm), and after weaning at postnatal day (P)28, the offspring was transferred to standard Plexiglas boxes (44×28×23 cm) in an air-conditioned room with a 12-h/12-h light-dark cycle and food and water ad libitum.

On P3-P4, pups were sexed and genotyped by PCR using tissue samples from tail tips [42]. The offspring showed the expected Mendelian ratio 1:1 for *Tsc2+/-* and wild-type genotypes.

For behavioral experiments, we used males (*n* = 36) from five different litters (littermates evenly distributed among experimental groups for counterbalanced design). Additional adult males from different litters, which underwent the same treatment as the main group, were used for the EEG testing (*n* = 18) and everolimus quantification (*n* = 16). Individual animals were arbitrarily distributed to experimental groups. Each rat had a unique identification code independent on group membership, permitting experimental blinding. Obviously ill or suffering animals would have been excluded from the study; however, no animal had to be excluded or died during the study.

All testing took place during the light phase of the daily cycle. Animals included in behavioral experiments were housed in groups of two or three. All experiments were approved by the Institutional Animal Care and Use Committee (Project of Experiments No. 66/2016) and complied with the Animal Protection Act of the Czech Republic, EU Directive (2010/63/EU). The study was not pre-registered.

### Group design and timing of procedures

**Fig. 1:**
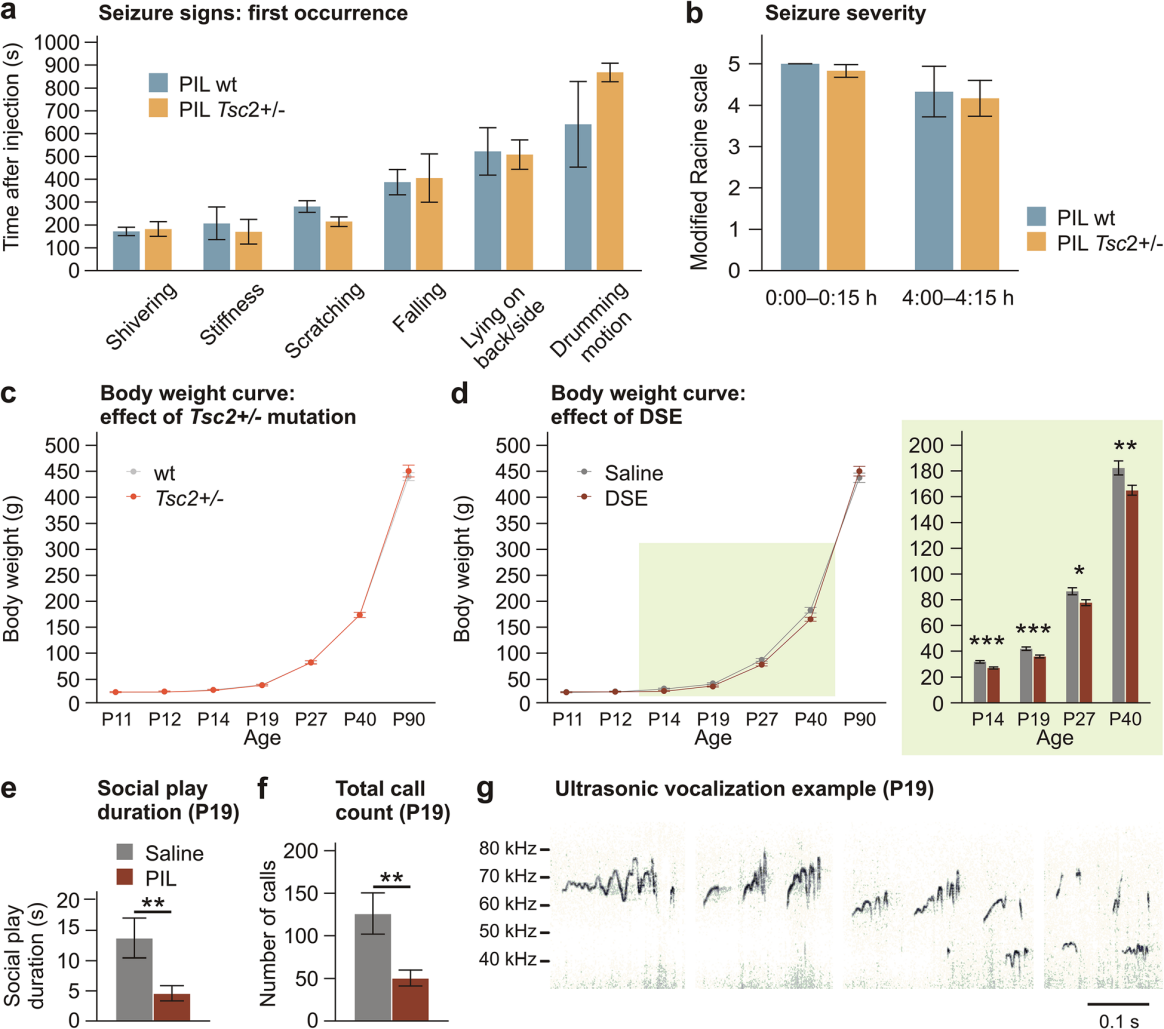
Induction of DSE and effects of *Tsc2+/-* and DSE on physical and behavioral development. **a**, **b** Pilocarpine injection induced signs of status epilepticus. Genotype of the pup had no influence on the time of the first occurrence of specific signs, and on seizure severity estimated using a modified Racine scale (*n* = 6 for wt, *n* = 6 for *Tsc2+/-*). **c** *Tsc2+/-* mutation did not affect body weight between P11 and P90 (*n* = 40 for wt, *n* = 30 for *Tsc2+/-*, DSE and saline pooled together). **d** Pilocarpine-induced DSE at P12 led to persistent growth retardation, which was detectable up to P40, but compensated in the adulthood (*n* = 33 for saline, *n* = 37 for DSE, *Tsc2+/-* and wt pooled together). **e**, **f** Pilocarpine-induced DSE led to reduction of social play and ultrasonic calling during interaction with an age- and treatment-matched conspecific at P19 (*n* = 8 pairs for saline, *n* = 9 pairs for pilocarpine, *Tsc2+/-* and wt pooled together). **g** Examples of ultrasonic calls emitted during social interaction at P19. Data are shown as means ± SEM. Three-way ANOVA with repeated measures and simple effects (**a**-**d**); *t*-test (**e**, **f**). *p < 0.05; **p < 0.01; ***p < 0.001

**Fig. 2:**
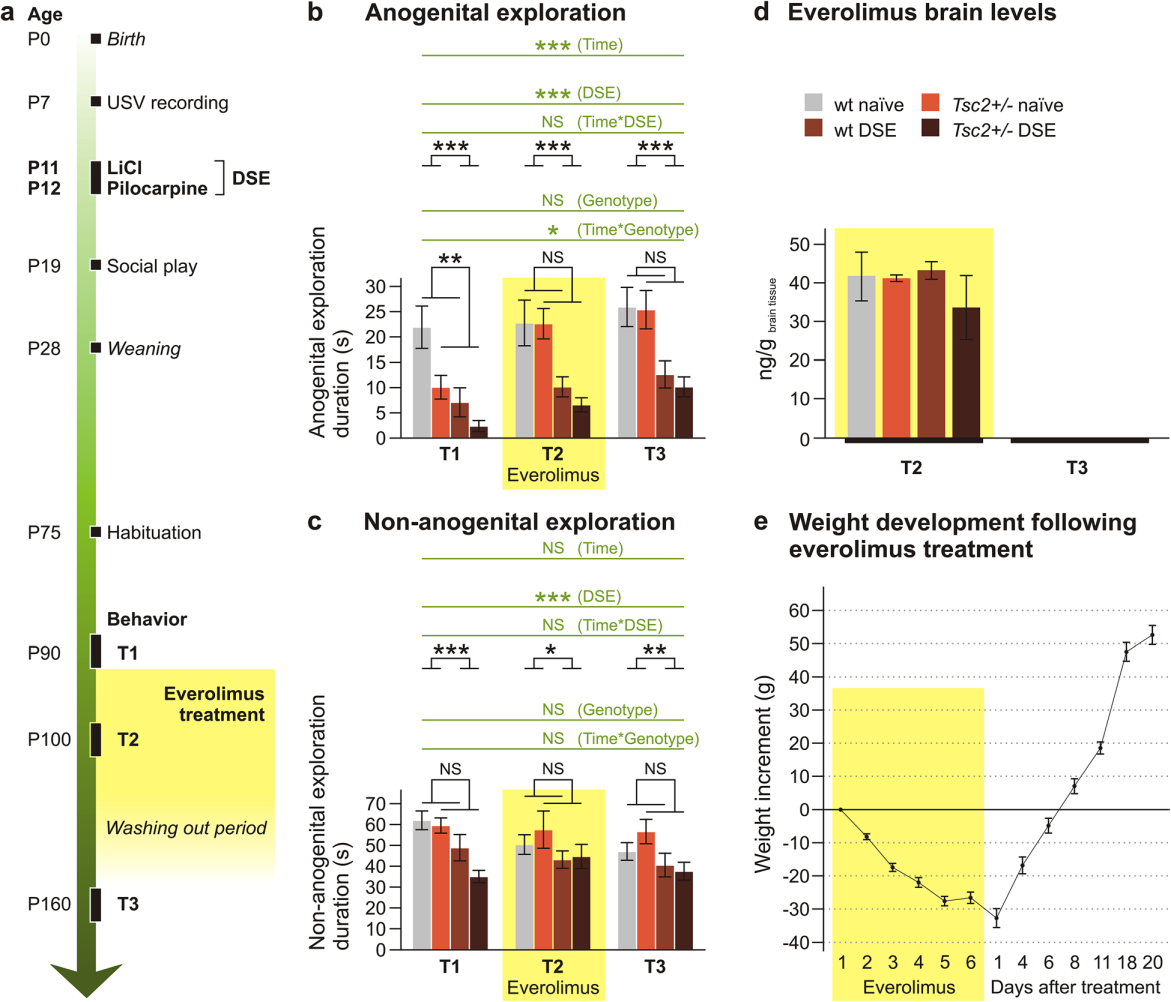
Effects of everolimus on social behavior in adult rats with *Tsc2+/-* and DSE. **a** Temporal order of behavioral tests and drug administration. **b**, **c** Duration of anogenital and non-anogenital social exploration before (T1), during (T2) and 8 weeks after (T3) everolimus administration (*n* = 10 for wt naïve, *n* = 7 for *Tsc2+/-* naïve, *n* = 11 for wt DSE, *n* = 8 for *Tsc2+/-* DSE). Anogenital exploration (**b**) was significantly reduced by DSE, and this effect was not changed by everolimus treatment. *Tsc2+/-* induced significant decrease in the baseline session (T1), which was ameliorated by everolimus medication (T2), and the improvement persisted even after washing-out of the drug (T3). Non-anogenital social exploration (**c**) was significantly impaired by DSE, but not by genotype. The changes induced by an early DSE experience in both types of social behavior were not affected by everolimus. **d** Concentration of everolimus in the brain after T2 testing was not affected by DSE or genotype. No everolimus was detectable at T3, 8 weeks after the last injection (at T2, *n* = 4 for wt naïve, *n* = 4 for *Tsc2+/-* naïve, *n* = 6 for wt DSE, *n* = 2 for *Tsc2+/-* DSE, at T3, *n* = 10 for wt naïve, *n* = 7 for *Tsc2+/-* naïve, *n* = 11 for wt DSE, *n* = 8 for *Tsc2+/-* DSE). **e** Two-week administration of everolimus led to marked weight loss in the experimental animals (*n* = 36). Data are shown as means ± SEM, significances are indicated by asterisks according to three-way ANOVA with repeated measures (in green) and two-way ANOVA (in black). NS *p* > 0.05; * *p* < 0.05; ** *p* < 0.01; *** *p* < 0.001

Sizes of experimental groups were calculated based on our data and experience from previous studies with the model [37–40, 43]. Group sizes were also influenced by the limited number of litters available from the mutant breeding line. Ultrasonic vocalizations (USV) as a measure of mother-seeking behavior were recorded at P7 in a proportion of animals (for detailed description, see Supplementary Methods). At P12, DSE or control treatment was administered. All animals were attributed to four experimental groups depending on genotype and DSE status: wild-type naïve, *Tsc2+/-* naïve, wild-type DSE, and *Tsc2+/-* DSE. At P19, a proportion of juvenile rats was behaviorally assessed as described below (Fig. 1). At the age of 3 months, the main experimental setup was initiated, focusing on the response to treatment with everolimus. It comprised assessment of behavior, epileptiform activity, and everolimus concentration in the brain. The experimental schedule consisted of three time points. First, rats underwent experiments under baseline (non-medicated) condition (T1), followed by 2 weeks of treatment with everolimus and a second round of experiments under medicated condition (T2). After 8 weeks of washing-out, a third round of experiments was conducted (T3). A graphical summary of the order of procedures and investigations can be found in Fig. 2a and Table 1.

**Table 1:** Design of the experiment in adults

Time points	Habituation	T1	Everolimus administration		Washing out period	T3
				T2		
**Procedure**	OF, EPM	Behavioral testing battery		Behavioral testing battery		Behavioral testing battery
**Age**	P74–P78	P81–P92		P95–P106		P158–P169
**Duration**	2 days	4 days		4 days	8 weeks	4 days
			12 days, 6 injections (every other day)			

*EPM* elevated plus maze test, *OF* open field test, *P* postnatal day

### Pilocarpine-induced developmental status epilepticus in pups

To simulate early developmental epilepsy, we used pharmacologically induced DSE. We decided to use lithium-pilocarpine-induced DSE at P12 instead of the previous paradigm of repeated kainic acid-induced DSE at P7 and P14 [39, 40]. The models are epileptologically very similar, but in a pilot experiment lithium-pilocarpine exhibited a stronger phenotype without mortality previously reported for kainic acid [39] (Fig. 1, Suppl. Fig. S1). The lithium-pilocarpine seizure model is well characterized and has been successfully used as a model of early epilepsy [44, 45].

Pilocarpine induction of DSE was performed following the protocol described by [46]. LiCl (127 mg/kg, i.p., dissolved in distilled water, purchased from Sigma Aldrich, Czech Republic, cat. no. L4408-100G) was applied to all pups at P11, to sensitize them to pilocarpine, and to facilitate seizures [47]. After 24 h (P12), half of the pups received a single i.p. injection of pilocarpine (35 mg/kg, injection volume 10 ml/kg, purchased from Sigma Aldrich, Czech Republic, cat. no. P6503-5G) and the others received 0.9% saline vehicle solution (naïve control). The state of the pups was checked by visual observation, a heating pad was used to prevent hypothermia, and additional saline was applied after the DSE procedure to prevent dehydration. With regard to brain development, the time span between P7 to P14 in rats is comparable with the first year of life in humans [48]. DSE in this period leads to life-long impairments of cognition and synaptic plasticity [49, 50]. Infantile spasms are a key component of early developmental epilepsy in TSC, associated with intellectual disability and ASD [15]. Chronic infantile spasms have been studied in rodents as genetic and as acquired models [51, 52]. There is however no single optimal model of infantile spasms, most have limitations, and many of the existing ones would not have been applicable for our study. We used DSE as an animal model of early epilepsy in TSC, although it is not a specific model of infantile spasms, in order to study differential effects of *Tsc2* haploinsufficiency and experience of status epilepticus restricted to early life [39, 40]. The DSE animal model resembles some aspects of early life epilepsy in TSC, in particular, the severe seizure activity and the long-term effects on behavior.

### Behavioral testing of juveniles

At P19, juvenile rats (*n* = 34) from the main experimental group were tested for spontaneous exploration of unknown environment and social behavior. In the open field test (OF), the juvenile was placed into the middle of a dimly illuminated (18 lux) Plexiglas box (40×35×21 cm) with fresh bedding and recorded for 3 min. Social interaction test was performed in the same box. After 5-min social isolation, two juveniles of the same genotype were put into the box simultaneously and recorded for 3 min. Social play, other social contacts, self-grooming, and digging were noted. Social play was the most distinctive behavioral category at this age, so we focused on it in the analysis. As it is a behavior in which both pups participate, every pair was taken as a single measurement. USV were recorded during SI sessions. The experimenter who handled the animal was always unaware of the experimental group assignment.

### Behavioral testing battery in adults

For the testing battery, we used 36 rats: wild-type naïve (*n* = 10), *Tsc2+/-* naïve (*n* = 7), wild-type DSE (*n* = 11) and *Tsc2+/-* DSE (*n* = 8). The order of animals was always randomized with respect to group membership. Due to limited throughput of the behavioral tests, the animals were divided into two cohorts shifted by 1 week. The behavioral testing battery was preceded by habituation sessions for open field and elevated plus maze apparatuses at P74–P78, to eliminate novelty effect from the subsequent testing. The behavioral testing battery conducted at T1, T2, and T3 covered several behavioral domains affected in TSC and ASD patients: social behavior and communication (social interaction, SI), anxiety (elevated plus maze test, EPM; light-dark test, LD), spontaneous locomotor activity (open field test, OF), learning and memory (Y-maze test; novel object recognition test, NORT). The design and temporal order of the tests is represented by Tables 1 and 2. The experimenter who handled the animal was always unaware of the experimental group assignment.

**Table 2:** Order of tests used in the behavioral testing battery

Phase	Habituation	Behavioral testing battery (T1, T2, T3)			
Day		Day 1	Day 2	Day 3	Day 4
**Test**	OF EPM	EPM LD	OF	NORT	SI Y-maze

*EPM* elevated plus maze test, *LD* light-dark test, *NORT* novel object recognition test, *OF* open field test, *SI* social interaction test

#### Social interaction test (SI)

In this test, two unfamiliar rats (non-cage mates) of the same age from the same group were interacting in the neutral, familiar environment of the open field arena, dimly illuminated (18 lux). Social isolation (10 min) preceded the interaction test, which itself took 10 min, but only the first 5 min were analyzed. The following parameters were evaluated: (a) social behavior: anogenital exploration, non-anogenital exploration, climbing on or pinning the social partner, following or approaching the social partner, play/fight, and evade; and (b) non-social behavior: self-grooming, freezing.

#### Elevated plus maze test (EPM)

The apparatus, made from light grey plastic, consisted of four arms (each 10-cm wide, 50-cm long), elevated 70 cm above the floor. Two arms opposite to each other were open, and the other two were surrounded by 30-cm high walls. The apparatus was illuminated by fluorescent tubes on the ceiling (480 lux in open arms, 85 lux in closed arms). Each animal was placed into the middle part facing into an open arm and recorded for 5 min. The animals were habituated to the apparatus by a 5-min session 1 week before T1 to avoid the effect of novelty. Time spent in the open arms was evaluated as a classical measure of anxiety. Looking down from the maze (a form of risk assessment behavior also termed head dipping or scanning in the literature) was counted whenever the head of the rat protruded over the edge of an open arm. This behavior has been shown to provide a measure of anxiety, perhaps linked to decision-making [53, 54]. The total number of arm visits was used as a measure of locomotor activity.

#### Light-dark test (LD)

The apparatus consisted of a dark compartment with black walls (25×25 cm) and a light compartment with white floor and transparent walls (50×25 cm; illumination 1000 lux), both divided by a gray wall disrupted by a 10×15 cm opening. It was manufactured to match the one used by Waltereit et al. (2011). The animal was placed into the dark compartment and recorded for 10 min. Time spent in the light compartment was recorded as a measure of anxiety [55].

#### Open field test (OF)

For the open field test, a square white chipboard arena (70×70 cm; illumination 1000 lux) was used. The animal was placed into the middle of the arena and left to freely explore for 10 min. Habituation to the arena was done 1 week before T1. Locomotor activity (total distance walked) was measured.

#### Novel object recognition test (NORT)

Novel object recognition test was done in the same apparatus as OF, but with a dim, indirect illumination (18 lux). It was done 24 h after the open field test to ensure familiarity of the animals with the environment. Two different pairs of identical objects of comparable size (glass jars full of pebbles and glass cuboid containers) were used. The objects were fastened by two-sided tape to the floor in the opposite corners (20 cm from the walls) and cleaned with water after each session. The animal was put into the central part facing an empty corner. In the initial sampling session, the rat was left free in the arena to explore two identical objects for 5 min. After 15-min retention interval, one object was replaced by a novel one and the animal was allowed to explore them for another 5 min (discrimination session). The use of objects as familiar/novel was counterbalanced to minimize the effect of any eventual preference of object or place, unrelated to the experimental stimulus. Discrimination index, as a measure of ability to differentiate between familiar and unfamiliar objects, was calculated as (*n*−*f*)/(*n*+*f*); where *n* is the time spent by the exploration of the novel object, while *f* is the time spent exploring the familiar object.

#### *Y*-maze test

Y-maze was used to study spontaneous alternation as a measure of working memory. The apparatus consisted of three identical arms (10-cm wide, 50-cm long, 30-cm high walls; labeled A, B, and C) made from white plastic. The rat was placed into the central part facing the arm A and was left free to explore the maze for 8 min. Spontaneous alternation (in %) was calculated as the number of correct triads of arm entries (ABC, BCA, CAB, ACB, CBA, BAC) divided by the number of all triads. The same arm entrance (AA, BB, CC) was counted when the rat came out of an arm to the center and returned back again to the same arm. Total arm visits were analyzed as an activity measure.

### Everolimus administration

Everolimus suspension was provided by Novartis (Basel, Switzerland) in a proprietary vehicle at 20 mg/ml. For application, it was diluted in 0.9% saline and injected i.p. at a dose of 1 mg/kg (injection volume 1 ml/kg), as described previously [40]. The drug was administered every other day for 12 days (6 injections in total), and the testing battery under medication (T2) was initiated during the second week of administration. All animals were injected by the active substance, and within-subject design was used to assess the effects on behavior. The drug was always applied in the afternoon, after behavioral testing. Body weight of rats was measured every day before the injection. Everolimus levels in the brain were not affected by rat genotype or history of DSE and remained stable in the period corresponding to the behavioral testing, as shown in Suppl. Fig. S3.

### Everolimus levels in the brain tissue

To address the question of everolimus levels in the brain during the behavioral tests, we harvested the brains from the main experimental group immediately after the T3 behavioral testing (8 weeks after the end of everolimus therapy). The rats were decapitated under isoflurane anesthesia. Additional rats of the same origin, age, and treatment (*n* = 16) were sacrificed at the end of the 14-day everolimus medication, corresponding to the end of T2.

To verify if everolimus concentrations during the T2 testing period were stable, we used a group of intact Wistar rats (RRID:RGD_13508588, Velaz Ltd., Czech Republic, *n* = 16). The rats undergoing the same everolimus treatment as the main experimental group were sacrificed either before the medication (baseline), or 24 h after the third or the sixth (final) everolimus injection, which corresponds to the beginning and the end of the T2 behavioral testing battery.

The rats were anaesthetized by isoflurane and sacrificed by decapitation, their brains quickly removed, cooled by dry ice, and stored at −80 °C until analysis. Approximately, 100 mg of rat brain tissue was transferred to Eppendorf tube; 100 mg of bullets for homogenization and 1 ml of acetonitrile were added to each sample. The samples were thoroughly vortexed and homogenized using the Bullet Blender Gold (Next Advance, USA) at 4 °C (15 min, speed 8). Then, the samples were centrifuged (10 min, 11.2 RPM) and the resulting supernatants were carefully pipetted to HPLC vials.

For HPLC-MS/MS analysis, UltiMate 3000 LC system (Thermo, USA) coupled with QTrap 6500 mass spectrometer (AB Sciex, Canada) was used. Chromatographic separation was performed on Kinetex F5 column, 2.1 x 150 mm, 1.7 μm (Phenomenex, USA). The mobile phases for gradient elution were 0.1 % formic acid + 5 mM ammonium formate in water (A) and methanol with 0.1 % formic acid (B). The flow rate of the mobile phase was 200 μL/min, and the column temperature was set at 30 °C. The MS/MS apparatus was operating in positive mode. A multiple reaction monitoring (MRM) method was developed with three transitions of *m*/*z* used for the detection of everolimus (975.6→908.5, 980.6→948.5, 980.6→775.5) and three transitions for the deuterated standard of the analyte (979.6→912.5, 984.6→952.5, 984.6→779.5). For data acquisition and management, Analyst software (RRID:SCR_015785) version 1.63 and MultiQuant 3.0.3 were utilized (AB Sciex).

### Stereotactic surgery

For EEG recordings, we used additional 18 male rats divided into the same groups: wild-type naïve (*n* = 5), *Tsc2+/-* naïve (*n* = 3), wild-type DSE (*n* = 4), and *Tsc2+/-* DSE (*n* = 6). The animals were stereotactically implanted at P80 with 14 gold-plated epidural electrodes (Mill-Max Mfg. Corp., product number 310-93-132-41-001, purchased from Farnell Czech Republic), under general isoflurane anesthesia (2.5%). Electrodes were implanted epidurally in homologous areas of the frontal, parietal, and temporal regions of the right and left hemispheres. Coordinates were taken from Paxinos rat brain atlas, RRID:SCR_006369 [56]: A +5 mm and L ±2 mm for the frontal association cortex (electrodes F3/F4), A +2.2 mm, and L ±3.2 mm for the primary motor cortex (electrodes C3/C4), A −3.8 mm and L ±2.5 mm (electrodes P3/P4), and A -4.5 mm and L ±4.5 mm (electrodes P5/P6) for the lateral parietal association cortex, A −3.6 mm and L ±7.2 mm for the temporal association cortex (electrodes T3/T4) and A −8.3 mm and L ±5.8 mm for the secondary auditory cortex (electrodes T5/T6). The reference electrode was implanted above the olfactory bulb and ground electrode subcutaneously in the occipital region. Electrode positions are indicated in Fig. 4. Electrodes and the connector were fixed to the skull with dental cement Dentalon (containing 1 g of active gentamicin per 100 g of Dentalon powder). After the surgery, rats were single-housed to prevent damage of the implant and left for 1 week to recover.

### EEG recordings

Four recordings were acquired from each animal. The first one was done 1 week after implantation (session 1 at P95—age corresponding to T1 behavioral experiments). Then, everolimus was administered for 2 weeks prior to the second recording (session 2 at P111, corresponding to T2 behavioral testing). Two more recordings were made during the washing-out period (session 3 at P124 and session 4 at P138), the latter corresponding to T3 behavioral testing.

Recording sessions were 42–55-min long (40 min of signal from each animal were analyzed) and were conducted in a box with bedding. The rats were able to move freely in the cage during EEG recording while connected to a data acquisition system.

Raw EEG signal was recorded using the BrainScope (M&I, Prague) BioSDA09 amplifier having a frequency band of 0.15–70 Hz. The system acquired data with a 16-bit depth, 7.63 nV/bit resolution (i.e., ∼130 bit/μV), and a dynamic range of ±500 μV. The data were recorded using a sampling rate of 1000 Hz.

### Data evaluation and statistical analysis

Sample sizes were adopted from previous behavioral experiments in the *Tsc2+/-* (Eker) rat [39, 40]. In some substantially laborious experiments (EEG recordings), the sample size was determined by the framework of experimental possibilities. With respect to our previous studies [39, 40], anogenital exploration was the primary outcome. No animal was excluded from the study.

During all behavioral tests, the animals were recorded by an overhead video camera located above the apparatus. BORIS software [57] was used for offline manual scoring of rat behavior; EthoVision software (RRID:SCR_000441, Noldus Information Technology, Wageningen, Netherlands) for automatic analysis of trajectory in OF. Ultrasonic calls were marked manually in the Audacity software (RRID:SCR_007198). EEG recordings were analyzed manually in BrainVision Analyzer 2.1 (RRID:SCR_002356), and all findings were video confirmed with behavior. All offline analyses were blinded.

Data were statistically evaluated using the IBM SPSS Statistics 25 (RRID:SCR_019096). Three-way analysis of variance (ANOVA) with repeated measures was used for the main behavioral testing battery. MANOVA, *t-*test and one-way ANOVA were used when applicable. The factors included in the analysis were genotype (*Tsc2+/-*) and DSE as between-subject measures and time as within-subject measure. The factor of time covered the course of everolimus medication (T1—baseline before treatment, T2—under everolimus medication, T3—after washing out). In case there was a significant genotype-time or DSE-time interaction, we used simple effects to specify the nature of the interaction and to identify the testing condition where the groups differed. The data was log-transformed to meet parametric assumption in case of non-normal distribution (indicated by Shapiro-Wilk test of normality). When transformation did not lead to normalization of the data (number of open arm visits in the elevated plus maze), the negative binomial model with log link function was used. Significance was accepted at *p* ≤ 0.05. When the assumption of sphericity was not met in ANOVA with repeated measures, we used Greenhouse-Geisser correction of df and *p* values.

Statistical analysis of the data was independently verified using GraphPad PRISM 5, RRID:SCR_002798 (two-way ANOVA and Bonferroni post hoc tests), also used to indicate significance in the figures. Data are shown as bar graphs with mean and SEM. With regard to the quantity of data combined in the figures, additional showing of individual data points resulted in overloaded presentations. Instead, full output tables of statistical results obtained from both types of statistical software can be found in Suppl. Tab. S1.

## Results

### Severity of pilocarpine-induced DSE is independent of pup genotype

Pilocarpine injection at P12 caused diarrhea and seizures accompanied by automatic scratching, drumming motions, tremors, limb extensions, postural problems, and wild running, which is consistent with signs described in the literature [58]. The signs appeared in characteristic order during seizure onset, and the time of their first occurrence was independent of pup genotype (Fig. 1a). Seizure severity was assessed by visual observation complemented by video recordings at two 15-min observation intervals, the first starting immediately after injection and the second starting 4 h after the injection. The signs were scored using a modification of the Racine scale. Clonic behaviors, such as shivering or forelimb clonus (“drumming “) counted as stage 3, while tonic behaviors (general stiffness, tail clonus, falls, or failure of righting reflex) counted as stage 5. Behaviors which could represent both seizure-related automatisms and normal behavior (i.e., scratching) only counted as stage 1. The highest stage observed in the 15-min interval for each animal was then counted. Seizure severity was comparable in *Tsc2+/-* and wild-type pups at both time points (Fig. 1b). This suggests that genotype did not play a role in the severity of the seizure. After 5–6 h, the symptoms receded in most individuals, and the pups were then returned to the mothers. No mortality was observed.

### Social behavior is altered by *Tsc2+/-* and pilocarpine-induced DSE during early development

Body weight of animals from all four experimental groups was monitored from P11 to P90. We found an obvious effect of age (*F*(1.261, 83.225) = 4207.379; *p* < 0.001; *η*_p_^2^ = 0.985), but also an interaction between age and DSE treatment (*F*(1.261, 83.225) = 4.092; *p* = 0.037; *η*_p_^2^ = 0.058). Genotype had no influence on weight. Simple effects showed significant differences between DSE and naïve groups at P14 (*p* < 0.001), P19 (*p* = 0.001), P27 (*p* = 0.015), and P40 (*p* = 0.010), with DSE animals exhibiting lower body weight. This indicates that DSE affected the physical development of the pups, with persistent retardation of growth only normalizing at early adulthood (Fig. 1c, d). Moreover, the effects of DSE on social behavior at P19 were examined. The total duration of social play was selected as the parameter most relevant to eventual autism-like deficits. The data were log-transformed because of non-normal distribution and analyzed by *t-*test. In pairs consisting of DSE pups, the social play was much less common: *T*(15) = 3.15, *p* = 0.007. Also, ultrasonic vocalizations (total call count) were much less common in DSE pups: *T*(15) = 3.035, *p* = 0.008 (Fig. 1e, f). Together, this confirms that DSE has a lasting impact on both physical and social developments of the pups.

In experimentally naïve P7 pups (prior to DSE), we observed an altered isolation-induced USV profile in *Tsc2+/-* pups, suggesting that *Tsc2+/-* also leads to early changes in vocal communication patterns (for detailed description, see Supplementary results, Fig. S2 and Suppl. Tab. S1).

### Everolimus reduces social behavior deficits in adult rats with *Tsc2+/-* but not after DSE

Young adult rats from all four experimental groups (wild-type naïve, *Tsc2+/-* naïve, wild-type DSE, *Tsc2+/-* DSE) were investigated in several behavioral paradigms under three different conditions: baseline before everolimus treatment (T1), during treatment with everolimus (T2), and after wash-out (T3).

In the social interaction test, the parameters of anogenital and non-anogenital social exploration were chosen for analysis (Fig. 2b, c, Supplementary Tab. S1). In anogenital social exploration, three-way ANOVA of T1–T3 revealed an effect of DSE, *F*(1, 32) = 23.909; *p* < 0.001; *η*_p_^2^ = 0.428, with rats with a history of DSE being less explorative. There was also an effect of time *F*(2, 64) = 16.078; *p* < 0.001; *η*_p_^2^ = 0.334. Importantly, there was a significant interaction between *Tsc2+/-* and time, *F*(2, 64) = 3.438; *p* = 0.038; *η*_p_^2^ = 0.097. A more detailed examination by simple effects analysis showed that *Tsc2+/-* genotype significantly decreased anogenital exploration at T1 (*p* = 0.036), but this deficit was no longer apparent at T2 and T3 (Fig. 2b). This indicates an effect of everolimus on anogenital social exploration impaired by *Tsc2+/-*, but not by DSE. In non-anogenital social exploration, three-way ANOVA of T1–T3 was only significant for the effect of DSE *F*(1, 32) = 12.285; *p* = 0.001; *η*_p_^2^ = 0.277. Two-way ANOVA of T1, T2, and T3 showed an effect only of DSE (Fig. 2c). Other parameters either did not differ between groups, or were too rare for analysis, and are not shown.

Importantly, we verified that everolimus concentration in brain tissue at T2 was not significantly affected by either DSE or *Tsc2+/-* (Fig. 2d). It was also stable during the T2 behavioral testing period and non-detectable after washing-out at T3 (Fig. 2d; Suppl. Fig. S3). Everolimus induced significant weight decrement, which was, however, quickly compensated after therapy discontinuation (Fig. 2e).

**Fig. 3:**
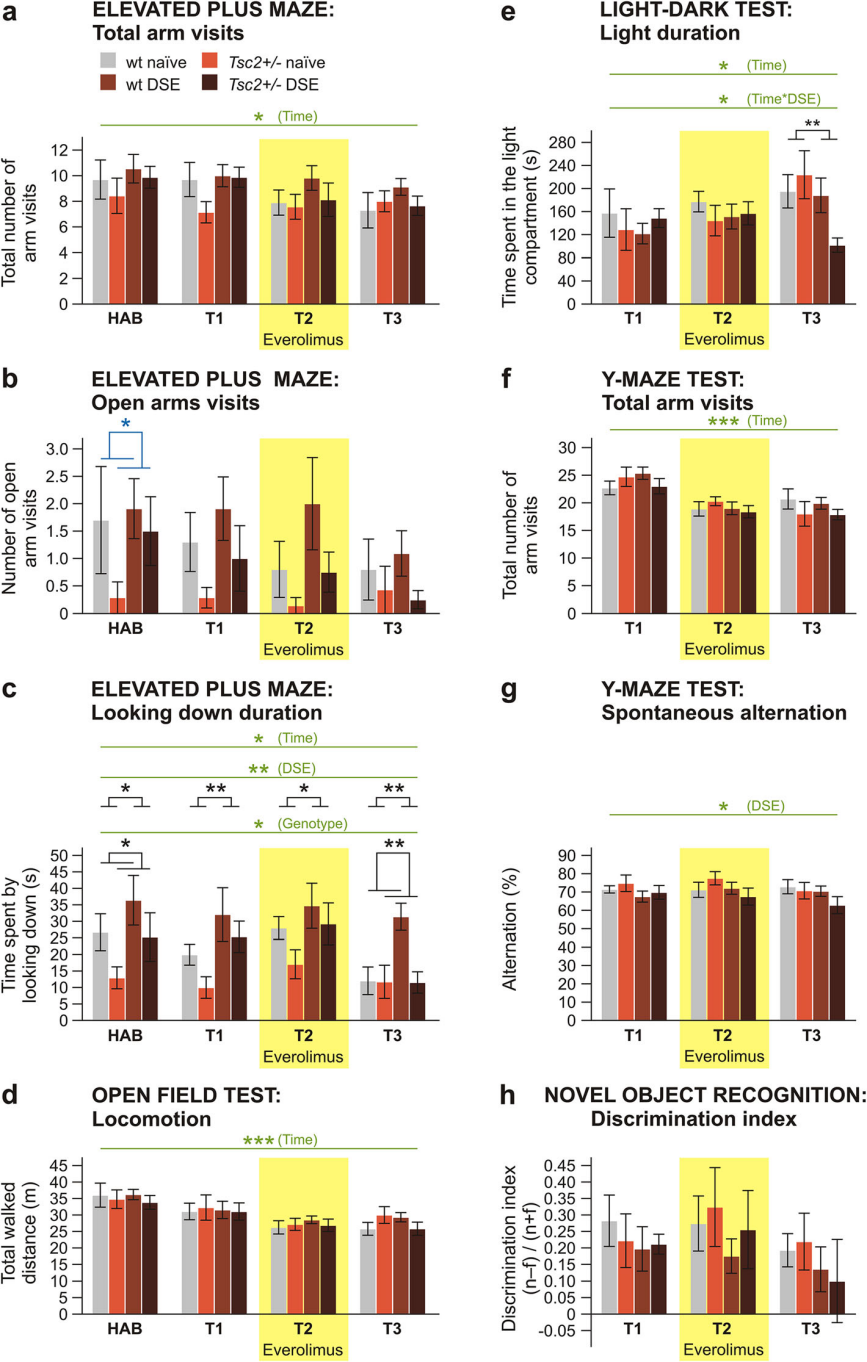
Effects of everolimus on non-social behaviors in adult rats with *Tsc2+/-* and DSE. In the elevated plus maze, overall activity measured as total arm visits (**a**) did not differ between groups. Anxiety measured by open arms visits (**b**) was not significantly different between groups in the experimental sessions, although in habituation, anxiety was increased in *Tsc2+/-* groups. Looking down duration (**c**) was increased by DSE and reduced by *Tsc2+/-* mutation. Locomotor activity in the open field (**d**), the time spent in the light compartment in the light-dark test (**e**) and general level of activity in the Y-maze (**f**) did not show differences between groups. On the other hand, spontaneous alternation in the Y-maze (**g**) was decreased in DSE groups according to repeated measures three-way ANOVA, revealing a working memory deficit (although the difference is not seen as significant in two-way ANOVAs for individual days). Novel object recognition (**h**) performance was not affected by either DSE treatment or genotype. Repeated measure analysis showed significant effect of testing condition in several parameters (**a**–**f**); however, in all cases, the behaviors evolved gradually in time in a manner suggesting habituation to the apparatuses or age-dependent changes, rather than everolimus medication as the cause (for all tests, *n* = 10 for wt naïve, *n* = 7 for *Tsc2+/-* naïve, *n* = 11 for wt DSE, *n* = 8 for *Tsc2+/-* DSE). Data are shown as means ± SEM, significances are indicated by asterisks according to three-way ANOVA with repeated measures (in green), two-way ANOVA (in black) or negative binomial model with log link (open arm visits, in blue). NS *p* > 0.05; * *p* < 0.05; ** *p* < 0.01; *** *p* < 0.001

Locomotor activity in the EPM, measured by total arm visits, was affected neither by *Tsc2+/-* nor by DSE. In three-way ANOVA, the effect of time was significant, *F*(3, 96) = 3.839; *p* = 0.012; *η*_p_^2^ = 0.107, corresponding to a gradual decrease of activity across sessions, probably driven by habituation of the animals (Fig. 3a). Open arm visits exhibited a strongly non-normal distribution, which was not improved by data transformation. Therefore, we used the negative binomial model with log link function and did not found a statistically significant effect of either DSE or genotype at T1, T2, or T3. However, the number of open arm visits was decreased in *Tsc2+/-* animals in the habituation session: 0.168 (95% CI, 0.029 to 0.972), *p* = 0.046 (Fig. 3b). In looking down duration, a three-way ANOVA showed a significant effect of DSE, *F*(1, 32) = 7.780; *p* = 0.009; *η*_p_^2^ = 0.196, with DSE rats spending more time looking down from the maze than their naïve counterparts. On the other hand, this behavior was reduced in *Tsc2+/-* rats, *F*(1, 32) = 6.205; *p* = 0.018; *η*_p_^2^ = 0.162. There was also an effect of time, *F*(2.168, 69.377) = 3.71; *p* = 0.026; *η*_p_^2^ = 0.104, suggesting gradual decrease. There was no interaction between the factors. Two-way ANOVA for individual sessions confirmed the effects of DSE at all time points. Effects of *Tsc2+/-* were present at habituation and T3, but not at T1 and T2 (Fig. 3c).

Locomotion of adult rats in the OF was neither affected by *Tsc2+/-* nor DSE. In three-way ANOVA, there was an effect of time (*F*(3, 87) = 18.233; *p* < 0.001; *η*_p_^2^ = 0.386), again suggesting a gradual decrease of locomotion either due to habituation or age-dependent changes in activity (Fig. 3d).

In LD, the time spent in the light compartment exhibited non-normal distribution and the data had to be log-transformed. In three-way ANOVA, there was an effect of time, *F*(1.443, 41.857) = 5.065; *p* = 0.019; *η*_p_^2^ = 0.149, suggesting gradual decrease of anxiety in time. There was a significant interaction between DSE and time, *F*(1.443, 41.857) = 4.329; *p* = 0.030; *η*_p_^2^ = 0.130. However, subsequent simple effect analysis did not find a specific significant difference on any testing day, only a trend at T3 (*p* = 0.071). Two-way ANOVA for individual sessions did not find specific differences at T1 and T2; however, animals that underwent DSE did spend less time in the light compartment at T3 (Fig. 3e). Bonferroni’s post hoc tests showed that it was due to the *Tsc2+/-* DSE group exploring the light compartment significantly less than any other group (Supplementary Tab. S1).

In the Y-maze, locomotor activity indicated by the total number of arm visits was only affected by time, *F*(2, 64) = 23.895; *p* < 0.001; *η*_p_^2^ = 0.427, with gradual decrease evidencing habituation to the environment. Working memory, measured by spontaneous alternation, was decreased in DSE animals, according to three-way ANOVA, *F*(1, 32) = 4.317; *p* = 0.046; *η*_p_^2^ = 0.119 (Fig. 3f, g).

Recognition memory in NORT, measured as discrimination index, was neither affected by *Tsc2+/-*, DSE nor time (Fig. 3h).

### *Tsc2*+/- and DSE induce epileptiform activity in adult rats which is not eliminated by everolimus

**Fig. 4:**
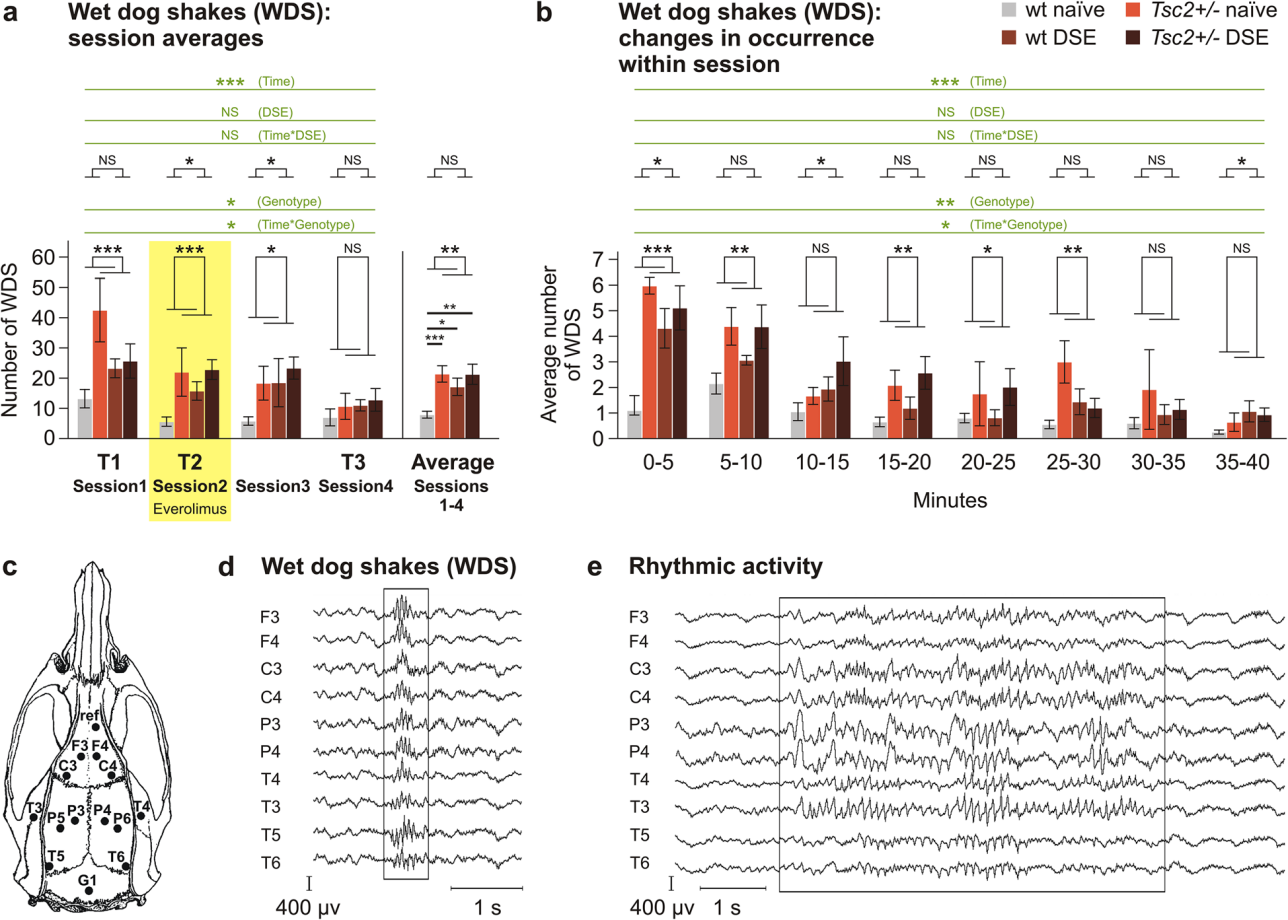
EEG recordings and epileptiform activity. **a** Number of wet dog shakes in *Tsc2+/-* and in DSE rats. In three-way ANOVA, there was a significant increase in WDS in *Tsc2+/-* rats and also generalized gradual decrease across sessions. In two-way ANOVA for data averaged across all sessions, the increase was significant for both *Tsc2+/-* and DSE. **b** Incidence of WDS also decreased during the course of a session. Placement of epidural electrodes is indicated in (**c**), and typical examples of epileptiform patterns are shown in **d** and **e**. Data are shown as means ± SEM (*n* = 5 for wt naïve, *n* = 3 for *Tsc2+/-* naïve, *n* = 4 for wt DSE, *n* = 6 for *Tsc2+/-* DSE), significances are indicated by asterisks according to three-way ANOVA with repeated measures (in green) and two-way ANOVA (in black) and Bonferroni post hoc test. NS *p* > 0.05; * *p* < 0.05; ** *p* < 0.01; *** *p* < 0.001

EEG in combination with video monitoring was recorded during T1 (session 1), T2 (session 2), 4 weeks later (session 3), and during T3 (session 4). EEG records showed normal and symmetric background activity and no signs of any generalized tonic-clonic seizures. However, epileptiform activity patterns were apparent in the data (Fig. 4, Supplementary Tab. S1).

During all sessions, patterns with a frequency of 15–20 Hz (mean duration 576±11 ms) were identified in the EEG (Fig. 4d), corresponding to wet dog shake (WDS) behavior on video recordings. Statistical analysis by three-way ANOVA of sessions 1–4 showed a significant effect of *Tsc2+/-*, *F*(1, 11) = 6.850; *p* = 0.024; *η*_p_^2^ = 0.384, with *Tsc2+/-* rats exhibiting more WDS. Simple effects showed that the difference between genotypes was significant in session 2 (*p* = 0.001) and session 3 (*p* = 0.019). The effect of DSE did not reach significance. There was an effect of time (*F*(3, 33) = 9.380; *p* < 0.001; *η*_p_^2^ = 0.460) with a robust gradual decrease of WDS events across sessions and interaction between time and *Tsc2+/-* (*F*(3, 33) = 3.586; *p* = 0.024; *η*_p_^2^ = 0.246). No specific effect of everolimus medication was apparent. Two-way ANOVA of individual sessions 1–4 mostly showed effects of both *Tsc2+/-* and DSE. Missing differences during session 4 could be due to a floor effect, as WDS decreased with time. We analyzed pooled data from sessions 1–4 to assess the overall occurrence of WDS. Two-way ANOVA and additional Bonferroni post hoc tests demonstrated individual and combined effects of both *Tsc2+/-* and DSE on increased WDS occurrence (Fig. 4a). To assess the temporal distribution of WDS within individual sessions, we pooled all sessions together and divided them into 5-min intervals. Three-way ANOVA showed a significant effect of *Tsc2+/-, F*(1, 14) = 13.007; *p* = 0.003; *η*_p_^2^ = 0.482, a strong effect of time, *F*(7, 98) = 19.223; *p* < 0.001; *η*_p_^2^ = 0.579, and interaction between time and *Tsc2+/-, F*(7, 98) = 2.202; *p* = 0.040; *η*_p_^2^ = 0.136. The number of WDS was initially high, rapidly decreasing during the course of the session. Two-way ANOVA showed a disseminated pattern of differences caused by *Tsc2+/-* and DSE (Fig. 4b).

During periods of behavioral immobility, we also noted long intervals (mean duration 3.5±0.2 s) of rhythmic EEG activity (frequency 8–10 Hz) (Fig. 4e), which occurred predominantly in *Tsc2+/-* rats (27 events compared to 1 in wt).

## Discussion

Our study aimed to investigate whether early epileptic seizure has an additional effect on the ASD-like phenotype in an animal model with *Tsc2* haploinsufficiency, and whether the *Tsc2+/-* and seizure-induced behavioral deficits differ in their responsivity to mTORi treatment. We found that the ASD-like social impairment induced by *Tsc2+/-* responded well to everolimus therapy, confirming our earlier work [38–40], while the behavioral consequences of DSE were not ameliorated by the drug.

The observed severity of pilocarpine-induced DSE was not affected by genotype, resembling similar seizure susceptibility to pentylenetetrazole described in *Tsc2+/-* and wild-type rats [38]. This is of importance because the initial insult was comparable in both genotypes, and the differences in adult animals have to be attributed to genotype effects later in development. We found that pilocarpine-induced DSE led to long-term disturbance of physical and social development, corresponding to changes documented in the literature [59]. We also noted qualitative changes in isolation-induced USV of 7-day-old pups bearing *Tsc2+/-* mutation. Altered vocalization patterns have been previously noted as an early manifestation of the ASD-like phenotype in *Tsc2+/-* mouse pups [41]. In adult rats, both DSE and *Tsc2+/-* mutation altered social behavior. While DSE led to general and persistent impairment of social exploration, *Tsc2+/-* specifically decreased anogenital social exploration in the baseline session. Anogenital and non-anogenital exploration behaviors seem to be quite independent from each other, so it may not be surprising to find the one to be more sensitive to genetic factors than the other [60]. As anogenital sniffing is important for individual recognition in rats [61], we consider it to be particularly sensitive to social deficits.

An ASD-like phenotype was previously reported in both epilepsy-naive *Tsc2+/-* (Eker) rats and wild-type rats after kainic acid-induced DSE [39, 40]. In a genetically comparable *Tsc2+/-* mouse model, decreased social interaction was found by Sato et al. [23], although an earlier study reported no social impairment [21]. Other genetic manipulations causing more severe *Tsc2* deficiency, or dysfunction of the TSC1-TSC2 complex, lead to abnormal social behaviors in mice [62, 63].

Importantly, a 2-week therapy with mTORi everolimus reversed the effect of *Tsc2+/-* on social behavior, with the beneficial effect persisting at least 2 months after discontinuation of the therapy. However, no such treatment response was observed in DSE animals. Although everolimus is known to suppress seizure frequency [17], it apparently did not ameliorate the persisting consequences of early developmental seizures. Our observations strongly suggest that *Tsc2+/-* and DSE may induce social impairments via different neurobiological mechanisms, with only the former being sensitive to mTOR inhibitors.

Both autism and epilepsy commonly affect emotionality. Increased anxiety after pilocarpine-induced DSE, described in the literature for both the EPM [64–66] and LD [58], was not replicated in our study. The exact timing of the status epilepticus might be crucial, as pilocarpine-induced seizure at older age actually *decreased* anxiety [67]. In the EPM, DSE animals spent more time looking down from the maze than naïve rats. Looking down from the maze may indicate exploratory behavior, inversely proportional to anxiety [53, 54]. However, open arm exploration as the main index of anxiety was not changed by DSE. We may also interpret looking down from the maze as a sign of risk assessment behavior, which was found to be elevated by DSE in other studies [64, 65].

Elevated anxiety was previously reported in the *Tsc2+/-* (Eker) rat [39], the findings in mouse models of TSC are contradictory [63, 68]. We saw elevated anxiety in the *Tsc2+/-* rats only in the EPM habituation session. It is possible that the anxiety phenotype was only apparent during the first experience with the task, as repeated testing has been shown to alter EPM behavior [69].

Regarding other domains of non-social behavior, DSE decreased working memory in the Y-maze. Impaired spatial learning was observed in rats after early postnatal seizures evoked by kainic acid, where it was underlain by a long-term loss of hippocampal plasticity, such as reduced capacity for long-term potentiation, reduced susceptibility to kindling and enhanced paired-pulse inhibition in the dentate gyrus [49]. A single early-life seizure in P7 rats resulted in impaired hippocampus-dependent short-term memory, but not spatial learning or recall. Presumably, the long-term cognitive impact of a single early-life seizure was limited largely to the hippocampus/prefrontal cortex [70]. It remains currently unknown if there are relations between these phenomena and the failure of everolimus to improve social behavior deficits following DSE. Locomotor behavior was unchanged in our experiments, suggesting normal sensorimotor function.

*Tsc1+/-* and *Tsc2+/-* mutations are known to induce epilepsy in humans, but so far, a spontaneous epilepsy phenotype has not been described in the *Tsc2+/-* rat or any comparable pre-clinical mouse models. However, previous studies assessed only overt behavioral signs of seizures [21, 38, 71, 72], which severely limited their sensitivity. Tschuluun et al. [73] measured EEG in intact and prenatally irradiated *Tsc2+/-* rats and detected no seizures, but their sample size was very small (*n*=2 for intact *Tsc2+/-* rats). Our EEG recordings show two classes of features which could be classified as epileptiform: wet dog shakes (WDS), possibly corresponding to focal limbic seizures [74], and intervals of rhythmic activity accompanied with immobility, both of which were more common in *Tsc2+/-* rats. WDS were, to a weaker extent than in *Tsc2+/-* rats, also observed in the adult rats with a history of DSE. Although epileptic status is a known trigger of epileptogenesis, very young animals seem to be more resilient to epilepsy initiation, and DSE before P17 did not lead to recurring seizures in adult rats [75], which is consistent with our observations. However, the occurrence of WDS decreased in time both between sessions and within a single session, especially in the rats carrying *Tsc2+/-* mutation, in a manner resembling habituation. This may suggest stress during the recording procedure as a possible trigger of epileptiform activity in the pre-disposed *Tsc2+/-* and DSE rats. Recently, Taylor et al. [76] have shown EEG signals in healthy rats mimicking absence seizures, which, however, can be terminated by external stimuli and therefore should not be considered true epileptiform activity. It is well possible that the rhythmic activity in our recordings was of similar nature, and it would be premature to classify it as epileptic without further verification. In addition, the limited number of animals in our EEG sample and rather short recording time limit the strength of our conclusions, but the observations are certainly interesting and deserve further examination. The present data do not show any effect of everolimus on EEG activity, but given the limitations by sample size and design, we must consider this conclusion tentative.

Despite the limitations, we showed that both *Tsc2+/-* and DSE rats exhibit distinct EEG changes, adding to the previously described increased sensitivity to flurothyl-induced seizures in irradiated animals [72] and kindling epilepsy in rats repetitively challenged with chemically induced seizures [38]. In clinical contexts, it remains unclear whether epilepsy in TSC patients is attributable to the *Tsc2+/-* mutation, is secondary to cortical tubers acting as epileptogenic foci [8] or may be a combination of both. Epileptiform patterns seen in the *Tsc2+/-* rat argue for the former, as the brains of *Tsc2+/-* rats are virtually tumor-free until advanced age [43].

The molecular mechanisms mediating the role of mTOR overactivation and epilepsy in the pathogenesis of ASD are only very partially understood. Aberrant synaptic protein synthesis induced by mTOR and MAPK overactivation was proposed to represent one possible pathway leading to autistic phenotypes [77]. Epilepsy sets in motion a cascade of events that include gene expression, sprouting of fibers, the establishment of new synaptic contacts, and thus persisting neuronal changes [78]. Our findings suggest *Tsc2+/-* and DSE to be pathophysiologically divergent, as only the former responded to mTORi treatment.

Returning to the clinical trial findings of mTORi in relation to TAND, the present study shows that the social impairment in a pre-clinical model responds to mTORi in a manner depending on its etiology: the effect of *Tsc2+/-* mutation is ameliorated by everolimus, while the effect of DSE is persistent—at least when treated for 2 weeks at adult age. If validated in other studies, our findings may have implications for clinical practice. Given that a significant proportion of individuals with TSC and ASD have early-onset epilepsy, our findings suggest that their ASD symptoms may only be partially responsive to mTORi. Our findings emphasize the importance of early seizure suppression as a fundamental approach against the development of ASD in TSC and in general [79–81]. Individuals with TSC and ASD who do not have comorbid epilepsy may represent a subgroup that could show more clear-cut benefit from mTORi treatment. Our study does not predict the effects of early developmental or long-term treatment with mTORi.

Finally, we acknowledge the limitations of any translational research and the challenges to recapitulate mutational and behavioral equivalence between human and rodent models. However, we used an established heterozygous animal model of TSC and used established paradigms of epilepsy and social behaviors, as used in pre-clinical research [82]. Our DSE paradigm with a single period of prolonged seizures was only an approximation of the situation in epilepsy patients, who experience recurring seizures throughout their lives. Further, it is unclear how valid pilocarpine-induced DSE is as a model for infantile spasms in TSC. It is however still remarkable that social behavior impairments in the epilepsy model could not be ameliorated by mTORi. Given that our study examined a 2-week treatment with everolimus in adult rats, it may not represent possible effects of early developmental and/or long-term treatment with mTORi. In addition, we did not assess the molecular pathways and mechanistic state of the mTOR signaling cascade in experimental groups and treatment stages directly. However, mTORi status in the brain was estimated by analyzing everolimus levels. Finally, we investigated here male animals only. In future research designs, it would be important to also study females. Despite the potential limitations, the findings presented here are novel and provide direct experimental evidence for differential contributions of molecular- and seizure-related mechanistic pathways to ASD-like phenotypes in TSC.

## Conclusion

We report here first, behavioral manifestations occurred early in the development of *Tsc2+/-* and DSE rats; second, both the presence of a *Tsc2+/-* mutation and a history of DSE decreased anogenital social exploration in adult rats, but only the deficit induced by *Tsc2+/-* was ameliorated by mTORi treatment; and third, we showed that both *Tsc2+/-* and DSE caused persisting epileptiform activity not eliminated by mTORi treatment. These findings suggest that both *Tsc2+/-* and DSE induce abnormalities in social behavior, but through different mechanisms. We propose that our findings may contribute to an explanation why mTORi treatment was not effective in TSC individuals with ASD where comorbid early-onset epilepsy is common.

## Supplementary Information

**Additional file 1:** **Fig. S1.** Impact of kainate- and pilocarpine-induced DSE on behavior in P27. **Fig S2.** Effect of Tsc2+/- on isolation-induced neonatal vocalization. **Fig S3.** Everolimus levels in the brain during administration. **Fig. S4.** Representative examples of EEG recordings. Supplementary results. Supplementary references

**Additional file 2: Supplementary Table S1. **Detailed statistical results from three-way ANOVA and from two-way ANOVA.

## Data Availability

The datasets used and/or analyzed during the current study are available from the corresponding authors on reasonable request.

## Abbreviations

ANOVA: Analysis of variance
ASD: Autism spectrum disorder
DSE: Developmental status epilepticus
EEG: Electroencephalogram
EPM: Elevated plus maze test
LD: Light-dark test
MAPK: Mitogen-activated protein kinase
mTOR: Mammalian (or mechanistic) target of rapamycin
mTORi: mTOR inhibitor/inhibition
NORT: Novel object recognition test
OF: Open field test
RRID: Research Resource Identifier (see scicrunch.org)
SEGA: Subependymal giant cell astrocytoma
SI: Social interaction test
TSC: Tuberous sclerosis complex
USV: Ultrasonic vocalization
WDS: Wet dog shake
wt: Wild-type
