# Supplementary material for: mTOR inhibitor improves autistic-like behaviors related to Tsc2 haploinsufficiency but not following developmental status epilepticus
Source: J Neurodev Disord. 2021 Apr 17;13:14. doi: 10.1186/s11689-021-09357-2 (PMC8052752; doi:10.1186/s11689-021-09357-2)
Supplement: Supplementary file 1 — Additional file 1: Fig. S1. Impact of kainate- and pilocarpine-induced DSE on behavior in P27. Fig S2. Effect of Tsc2+/- on isolation-induced neonatal vocalization. Fig S3. Everolimus levels in the brain during administration. Fig. S4. Representative examples of EEG recordings. Supplementary results. Supplementary references [file 11689_2021_9357_MOESM1_ESM.docx]

**Supplement**

**mTOR inhibitor improves autistic-like behaviors related to Tsc2 haploinsufficiency but not following developmental status epilepticus**

Tomas Petrasek^1^, Iveta Vojtechova^1,2^, Ondrej Klovrza^1,3^, Klara Tuckova^1,4^, Cestmir Vejmola^1^, Jakub Rak^1^, Anna Sulakova^1^, Daniel Kaping^1^, Nadine Bernhardt^5^, Petrus J de Vries^6^, Jakub Otahal^7^, Robert Waltereit^8,9^

*^1^National Institute of Mental Health, Klecany, Czech Republic*

*^2^First Faculty of Medicine, Charles University, Prague, Czech Republic*

*^3^Second Faculty of Medicine, Charles University, Prague, Czech Republic*

*^4^Faculty of Science, Charles University, Prague, Czech Republic*

*^5^Department of Psychiatry, University Hospital and Medical Faculty Carl Gustav Carus, Technical University of Dresden, Germany*

*^6^Division of Child & Adolescent Psychiatry, University of Cape Town, Cape Town, South Africa*

*^7^Department of Developmental Epileptology, Institute of Physiology CAS, Prague, Czech Republic*

*^8^Department of Child and Adolescent Psychiatry, University Hospital and Medical Faculty Carl Gustav Carus, Technical University of Dresden, Germany*

*^9^Department of Child and Adolescent Psychiatry, University Medical Center Göttingen, Germany*

Corresponding Authors:

PD Dr. med. Robert Waltereit

Department of Child and Adolescent Psychiatry

University Medical Center Göttingen

Von-Siebold-Str. 5, 37075 Göttingen

Germany

Telephone: +49 551 39 8958

Email: [robert.waltereit@med.uni-goettingen.de](mailto:robert.waltereit@med.uni-goettingen.de)

Tomas Petrasek, PhD

National Institute of Mental Health

Topolova 748, 250 67 Klecany

Czech Republic

Telephone: +420 283 088 246

Email: [tomas.petrasek@nudz.cz](mailto:tomas.petrasek@nudz.cz)

**SUPPLEMENTARY METHODS**

**Comparison of kainate- and pilocarpine-induced DSE models**

In the previous studies [1,2], we have used kainic acid-induced DSE model. However, the pilocarpine-induced DSE model has previously been reported to have similar neurobiological impacts, with the advantage of negligible mortality [3]. Therefore, we decided to compare the two DSE models prior to the main experiment, and choose the better one, in terms of pup mortality and social behavior phenotype. In wild-type Long-Evans rats of both sexes (n = 27) from two different litters, we applied either pilocarpine (n = 7), or kainate (n = 11), or control (saline, n = 9) injection.

Kainic acid (purchased from Sigma Aldrich, Czech Republic, cat. no. K0250-10MG) was injected twice to each pup, at P7 (2 mg/kg) and at P14 (4 mg/kg). This application protocol was employed previously in the studies by [1,2]. However, we used a slightly lower dose at P7 than the previous studies, to avoid risk of mortality. Pilocarpine administration followed the same protocol as described below, which was then used for the main experiment.

Weights of the pups were monitored prior and after the DSE. Following DSE, the pups were tested in open field (40⨯35⨯21 cm box with bedding) at P27. Social interaction test was conducted in the same box, always using two pups from the same treatment group. Social play behavior and ultrasonic vocalizations were noted. For behavioral and ultrasonic observation and subsequent statistical analysis, a pair was taken as a single measurement, because social play involves both animals, and ultrasonic calls cannot be attributed to a specific individual.

**Neonatal ultrasonic vocalization**

Isolation-induced ultrasonic vocalizations (USV) were recorded as a measure of mother-seeking behavior in pups (n = 55) at P7 (prior to DSE treatment). Each pup was put into a small Plexiglas box (30⨯18⨯14.5 cm) containing fresh bedding and left alone for 45 seconds while vocalizations were recorded using Ultramic 250k microphone (Dodotronic, Italy, cat. no. SKU: UM250K) and Audacity 2.2.0 software. In the analysis, two categories were differentiated: simple neonatal calls and neonatal calls with frequency steps. Number and total duration of calls were evaluated. Duration of bouts (trains of successive vocalizations separated by less than 750 ms gaps) was also measured, and median call duration noted for each individual.

**SUPPLEMENTARY RESULTS**

**Comparison of kainate- and pilocarpine-induced DSE models**

Visual observation of the DSE procedure suggested kainate-induced seizures to be less pronounced and more intermittent than pilocarpine-induced seizures. In the open field test at P27, one-way ANOVA didn’t show any differences in locomotion between kainate, pilocarpine and saline groups: F(2) = 0.497, P = 0.615 (Fig. S1c). During social interaction at P27, social play was common in control animals. Ultrasonic recordings showed that low-frequency vocalizations (similar to neonatal calls or adult anxious vocalizations) did not occur, but high-frequency calls were rather common. Although those could be further sub-divided [4], we took them as a single category. Kainate treatment affected neither the duration of social play, nor the number of ultrasonic vocalizations. On the other hand, the pups after pilocarpine-induced DSE exhibited a decrease in both parameters (Fig. S1a, S1b).

**
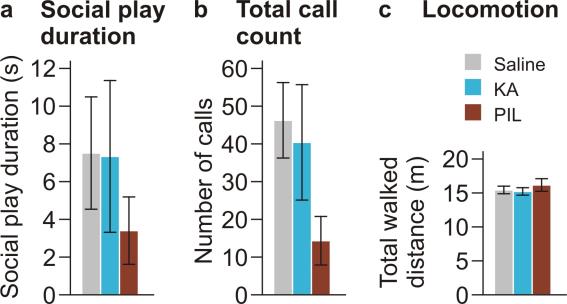
**

***Figure S1: Impact of kainate- and pilocarpine-induced DSE on behavior in P27****During interaction of two age- and treatment-matched pups at the age of P27 (pre-weaning), social play behavior (a) and total number of ultrasonic vocalizations (b) were decreased in the pups which underwent pilocarpine-induced DSE (PIL), but not kainate-induced DSE (KA). Locomotor activity in unfamiliar environment (c) was not affected by the treatment (n = 9 for saline, n = 11 for kainic acid, n = 7 for pilocarpine). Data are shown as means ± SEM.*

**Alterations in ultrasonic vocalization in *Tsc2+/-* pups**

All 7-day-old pups exhibited isolation-induced vocalizations with the fundamental frequency ranging between 35 and 45 kHz, often with harmonic frequency around 70 – 80 kHz, mostly occurring in series (bouts) of variable duration (Fig. S2d-f). Less common “adult-like” calls with higher fundamental frequencies (up to 70 kHz) were also noted in some individuals, but not included in the analysis. Their abundance didn’t seem linked to genotype. We analyzed the total number of isolation-induced calls, number of isolation-induced calls with frequency steps (stepped calls) and median bout duration. The latter two parameters had non-normal distribution and had to be log-transformed. Analysis by MANOVA didn’t show overall significance. Tests of Between-Subjects Effects showed that *Tsc2+/-* individuals exhibited lower number of vocalizations with frequency steps (p = 0.026) and decreased median bout duration (p = 0.016). However, these observations have to be interpreted with caution, as the effect size was low (Ƞ_p_^2^ = 0.094 and 0.109, respectively), and in the latter case the statistical outcome was affected by the presence of an outlier. Nevertheless, the impact of *Tsc2* mutation on neonatal vocalization clearly deserves further attention, as it suggests early onset of the behavioral phenotype (Fig. S2a-c).


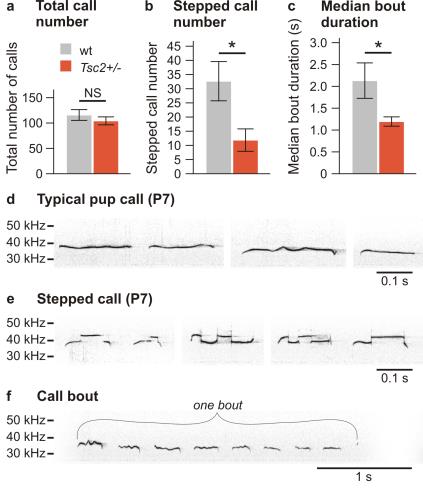


***Figure S2: Effect of Tsc2+/- on isolation-induced neonatal vocalization****(a) Tsc2+/- mutation didn’t affect the total number of isolation-induced vocalizations in P7 pups. (b) However, Tsc2+/- mutant individuals exhibited lower number of isolation-induced vocalizations with frequency steps and (c) their vocalization bouts were significantly shorter (n = 33 for wt, n = 22 for* Tsc2+/-*). (d-f) Typical examples of isolation-induced calls and one bout.* *Data are shown as means ± SEM. *p < 0.05*

**Everolimus levels in the brain tissue**

In the pilot experiment in intact wild-type rats, everolimus was not detected in the baseline (unmedicated) group. In brains harvested 24 h after the third and 24 h after the sixth injection of everolimus, concentrations of the drug were comparable, suggesting a stable therapeutic level during the T2 behavioral testing (Fig. S3).

*
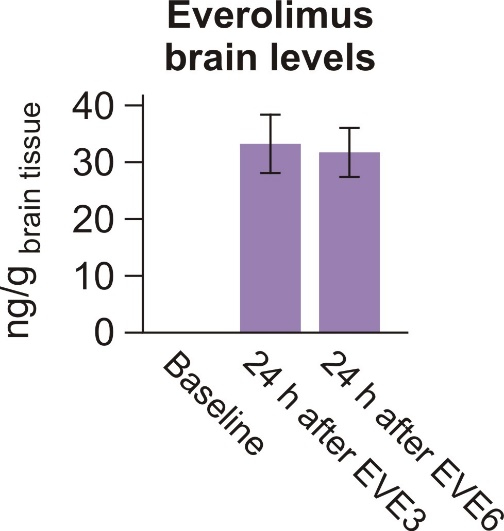
*

***Figure S3: Everolimus levels in the brain during administration****Brain levels of everolimus were stable in the interval between the third (EVE3) and the sixth (EVE6) injection, when the T2 behavioral testing occurred (n = 4 for baseline, n = 6 for EVE3 and n = 6 for EVE6). Data are shown as means ± SEM.*

**Representative examples of EEG recordings**

In addition to the quantitative data on epileptiform activity, we present here representative examples of wet-dog shake (WDS) EEG patterns from all sessions of all four experimental groups (Fig. S4).


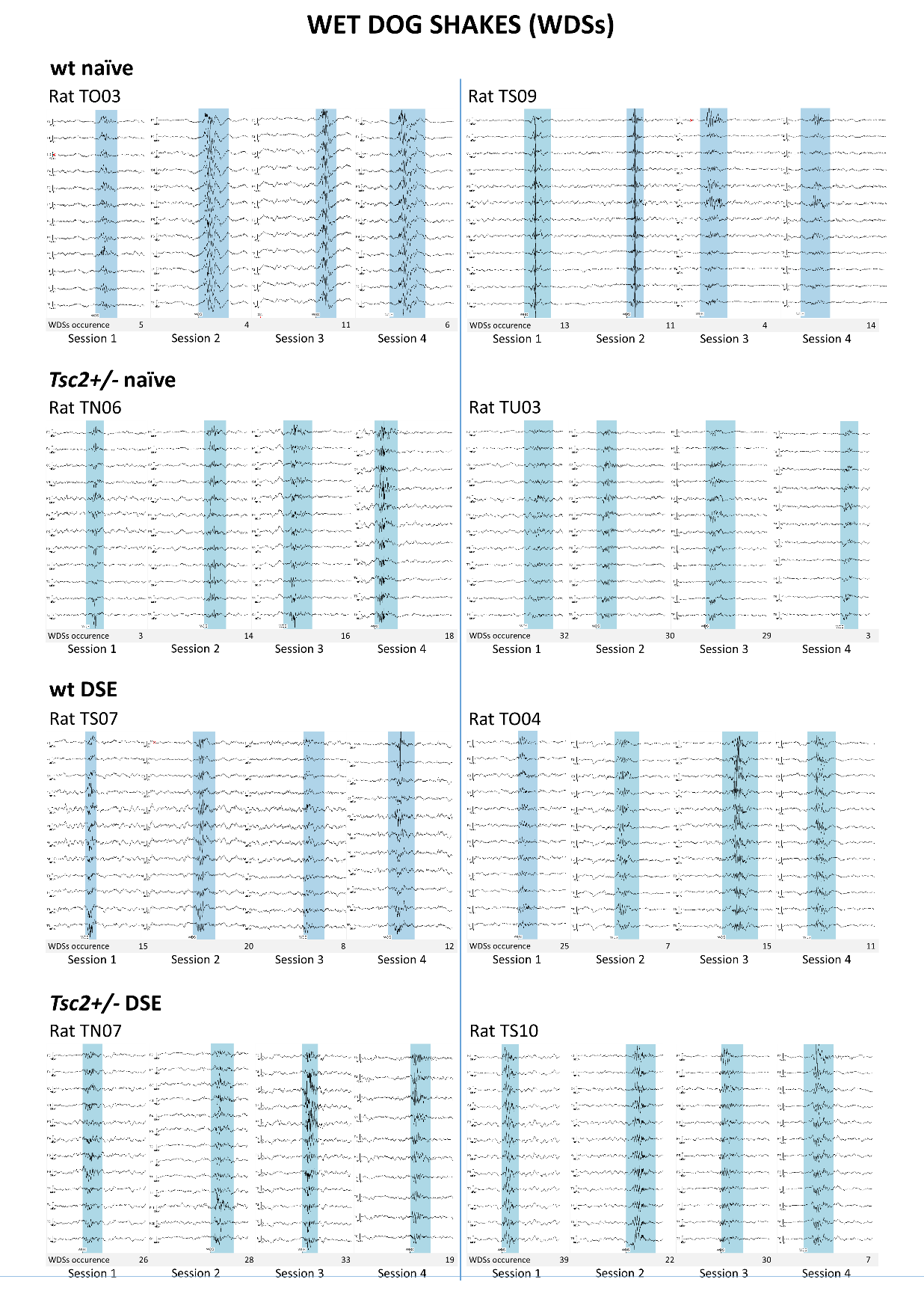


***Figure S4: Representative examples of EEG recordings***

Shown are WDSs from EEG recordings from all four sessions. The number of WDSs occurring within the respective session is indicated.

**SUPPLEMENTARY REFERENCES**

1. Waltereit R, Japs B, Schneider M, De Vries PJ, Bartsch D. Epilepsy and Tsc2 haploinsufficiency lead to autistic-like social deficit behaviors in rats. Behav Genet. 2011;41:364–72.

2. Schneider M, de Vries PJ, Schönig K, Rößner V, Waltereit R. mTOR inhibitor reverses autistic-like social deficit behaviours in adult rats with both Tsc2 haploinsufficiency and developmental status epilepticus. Eur Arch Psychiatry Clin Neurosci. 2017;267.

3. Reddy DS, Kuruba R. Experimental models of status epilepticus and neuronal injury for evaluation of therapeutic interventions. Int J Mol Sci. 2013;14:18284–318.

4. Wright JM, Gourdon JC, Clarke PBS. Identification of multiple call categories within the rich repertoire of adult rat 50-kHz ultrasonic vocalizations: effects of amphetamine and social context. Psychopharmacology (Berl). Germany; 2010;211:1–13.
